# Using picoliter droplet deposition to track clonal competition in adherent and organoid cancer cell cultures

**DOI:** 10.1038/s41598-023-42849-w

**Published:** 2023-11-01

**Authors:** Selami Baglamis, Vivek M. Sheraton, Debora Meijer, Haibin Qian, Ron A. Hoebe, Kristiaan J Lenos, Max A. Betjes, Max A. Betjes, Sander Tans, Jeroen van Zon, Louis Vermeulen, Przemek M. Krawczyk

**Affiliations:** 1 Laboratory for Experimental Oncology and Radiobiology, Center for Experimental and Molecular Medicine, Amsterdam UMC Location University of Amsterdam, Meibergdreef 9, 1105 AZ Amsterdam, The Netherlands; 2 Cancer Center Amsterdam, 1081 HV Amsterdam, The Netherlands; 3 Oncode Institute, 3521 AL Utrecht, The Netherlands; 4 Amsterdam Gastroenterology Endocrinology Metabolism, 1105 AZ Amsterdam, The Netherlands; 5 Institute for Advanced Study, University of Amsterdam, 1012 WX Amsterdam, The Netherlands; 6 Department of Medical Biology, Amsterdam University Medical Centers (location AMC), University of Amsterdam, Meibergdreef 9, 1105 AZ Amsterdam, The Netherlands; 7 AMOLF, 1098 XG Amsterdam, The Netherlands

**Keywords:** Cancer, Cell biology

## Abstract

Clonal growth and competition underlie processes of key relevance in etiology, progression and therapy response across all cancers. Here, we demonstrate a novel experimental approach, based on multi-color, fluorescent tagging of cell nuclei, in combination with picoliter droplet deposition, to study the clonal dynamics in two- and three-dimensional cell cultures. The method allows for the simultaneous visualization and analysis of multiple clones in individual multi-clonal colonies, providing a powerful tool for studying clonal dynamics and identifying clonal populations with distinct characteristics. Results of our experiments validate the utility of the method in studying clonal dynamics in vitro*,* and reveal differences in key aspects of clonal behavior of different cancer cell lines in monoculture conditions, as well as in co-cultures with stromal fibroblasts.

## Introduction

Cancer heterogeneity largely determines tumor growth, metastatic behavior and, ultimately, treatment resistance^1^. At its root, it is a consequence of genome instability that leads to the emergence of tumor sub-populations, characterized by diverse, and often non-overlapping mutational profiles^2^. As these, in turn, give rise to different phenotypic manifestations, including those driving drug resistance, a single cell can evolve into a population of cells displaying a plethora of resistance mechanisms to many modern cancer therapies^3, 4^.

Intratumoral population dynamics is a product of complex relations, including competition, cooperation and ecological interactions, between the different sub-populations (clones), as well as between the clones and the tumor microenvironment^5–7^. As these relations ultimately drive the evolutionary processes, they are the subject of intense investigation using in silico^8^, in vitro^9, 10^ ex-vivo and in vivo^11, 12^ approaches. In many cases, biological experiments are designed to extract key system parameters, which are subsequently modeled using computational approaches^13–15^.

While complex biological models, such as those utilizing experimental animals or even in-human interventions, generally mirror the real-world tumor dynamics more closely^11, 16–19^, the resulting models are often overparameterized and thus difficult to interpret. As a consequence, simplified experimental approaches have also been proposed and employed to study various aspects of clonal dynamics in vitro^20^, with cellular barcoding being a key strategy^20–22^. Relying on different genetically encoded tags, activated either permanently or on-demand, barcoding allows tracking of cell progeny across multiple generations and over extended periods of time. Genetic barcoding involves incorporation of short, unique DNA sequences (barcodes) into the genomes of (cancer) cells; deciphering cell lineage information with such tags is then generally accomplished with help of DNA sequencing after cell fixation, or from cell culture supernatants^22–24^. Optical tagging relies, instead, on incorporation of DNA sequences encoding fluorescent proteins of different emission wavelengths, expression of which can be monitored using flow cytometry or (live-cell) microscopy^18, 22, 25^. This latter approach is uniquely suitable not only for quantifying the sizes of clonal populations, but also for capturing their dynamics in two or three dimensions plus time^26–28^.

Here we propose a simple approach to analyzing clonal population dynamics in adherent 2D cell cultures and in 3D organoid cultures, using fluorescent optical tags derived from the widely-used lentiviral gene ontology (LeGO) vectors vectors^18, 25, 29, 30^. We have modified these tags by fusing them with either histone H2B or nuclear localisation signal (NLS) for facilitating localization of individual cells using 2D and 3D microscopy. Using various cancer cell lines stably expressing these tags, we then generate small, multi-clonal colonies, using a pico-liter droplet dispenser, and track the growth of these colonies, and the clones they consist of, in time, using fluorescence microscopy.

Results of our proof-of-concept experiments reveal differences in spatio-temporal clonal dynamics of different cancer cell lines, and effects of the presence of non-transformed cells (fibroblasts), which appear to stimulate clonal diversity in two-dimensional cultures. We then analyze clonal dynamics in 3D cultures of human colorectal cancer (CRC) cells harboring different mutations that are relevant in CRC etiology. In summary, this study demonstrates that our method can be applied to investigating clonal dynamics in various 2D and 3D settings in vitro and, potentially, ex vivo.

## Results

### Generating nuclear gene ontology (LeGO) tag derivatives

**Figure 1:**
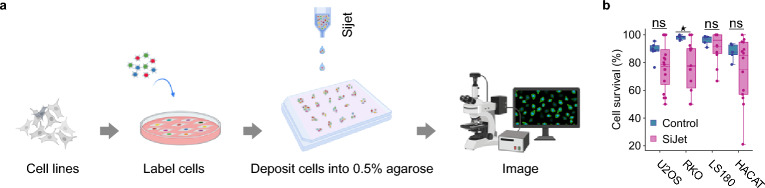
Schematic overview and cell survival after picoliter deposition. (**a**) Schematic overview of the experimental pipeline, created with BioRender.com. (**b**) The effect of picoliter droplet deposition on cell survival. The indicated cell lines have been plated under control conditions, or subjected to the SiJet picoliter deposition procedure, and their survival has been monitored using time-lapse microscopy. Each data point in the control group represents an average survival of at least 10 cells. In the SiJet group, each data point represents a single colony.

While studying clonal dynamics in bulk cell cultures can yield important insights^31, 32^, here we decided to focus on single, multi-clonal (i.e. originating from multiple individual cells rather than from a single cell) colonies, approximating simplified individual tumors, and study them in spatial isolation from other colonies. To enable visualization of individual cells and clonal cell progeny, we adapted the lentiviral gene ontology (LeGO) optical tags^18, 25^ and fused them, via a flexible linker, to the human nuclear-localization signal (NLS) or to human histone H2B, resulting in LeGO-NLS and LeGO-H2B, respectively. Both methods ensured nuclear localization of the LeGO tags, increasing the signal-to-noise ratio in fluorescence imaging and, in the case of H2B fusions, allowing us to image the cells throughout the cell cycle. Both NLS and H2B fusions are among the most widely used approaches that have few or no observable effects on the host cell^33, 34^. The experimental setup is depicted in Fig. 1.

### Generating multi-clonal colonies of adherent cells

**Figure 2:**
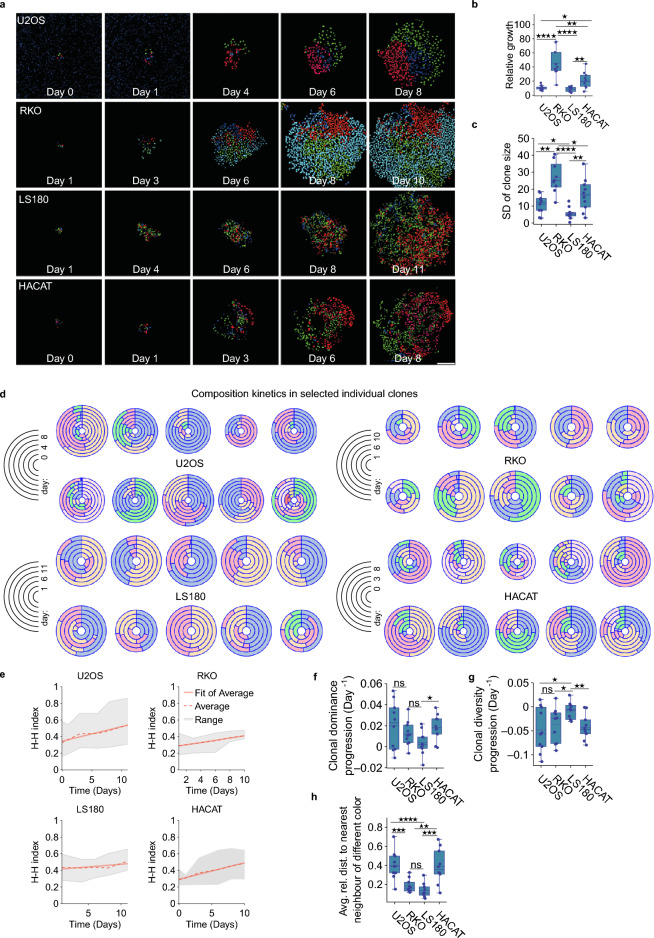
Clonal growth dynamics. (**a**) Representative images of selected colonies at the indicated time points; scale bar: 200 µm. (**b**) Relative growth of colonies generated from the indicated cell lines. Each data point represents the number of cells in a single colony on day 6 of the experiment, normalized to the number of cells in the respective colonies at day 1 of the experiment. (**c**) Standard deviation of clone sizes in colonies analyzed in (**b**), representing clone growth heterogeneity. (**d**) Visualization of size changes over time in selected individual clones of the indicated cell lines. The concentric rings represent time, with earliest time points drawn at the core of each plot. (**e**) Changes in the Herfindahl–Hirschman index, a measure of clonal size dominance, for different cell lines in mono-culture. The shaded areas indicate the range of the index values, dotted and continuous lines represent the mean and linear fitted values of Herfindahl–Hirschman index at different timepoints, respectively. (**f**) Box plot comprising the slope values of the linear fit of Herfindahl–Hirschman index values vs time data. (**g**) Box plot comprising the slope values of the linear fit of Shannon index values (a measure of clonal diversity) vs time data. (**h**) Clonal intermixing of indicated cell lines on day 6.

To study the clonal dynamics in adherent cells, we first generated colorectal cancer cell lines RKO and LS180, human bone osteosarcoma epithelial cell line U2OS and human epidermal keratinocyte line HACAT, stably expressing various random combinations of LeGO-NLS tags^18^ (Fig. S1), resulting in a range of colors confined to cell nuclei (Fig. 2a).

To generate compact, multi-clonal colonies, we used SiJet, an automatic dispenser that delivers mechanical shock to a small liquid container, which ejects, through a nozzle, picoliter-range droplets containing suspended cells. The droplets were deposited into cell culture vessels pre-filled with 0.5% low-melting-point agarose (dissolved in medium) at ~ 37 °C. The agarose prevented the cells in deposited colonies from drifting apart due to the random brownian motion or other microcurrents in the cell culture vessel, but was removed and replaced with regular cell-culture medium after the cells attached to the bottom of culture vessels (~ 2h). This procedure resulted in multi-clonal colonies containing ~ 2–10 cells, generally expressing different combinations of the LeGO-NLS tags and thus characterized by different colors, which enabled us to track the progeny of each cell over time (Fig. 2a). The procedure appeared to have a limited detrimental effect on cell viability, as estimated by the fraction of surviving clonogenic cells (Fig. 1b).

### Image analysis pipeline

To analyze the clonal dynamics in the two-dimensional colonies, we developed a custom image analysis pipeline capable of detecting and segmenting cell nuclei, and distinguishing between the different colors (Fig. S2a–f). We validated the pipeline by comparing the results of automatic cell detection and color classification to the results of human-performed operations. We found a relatively good correspondence in most cases, with the automatic algorithm generally detecting fewer cells than the human operator (Figs. S2g, S3 and S4).

### Analyzing clonal dynamics in adherent colonies

After establishing the optically tagged cell lines and imaging pipeline, we applied our colony deposition method to study the clonal dynamics in different cancer cell lines. To this end, we imaged the colonies over the period of 8–10 days (Fig. 2a), and quantified the changes in sizes of different clones, represented by colony subpopulations with a distinct color (Fig. 2b–d). As some colonies initially contained more than one cell of a given color, we normalized clone sizes by dividing them by the initial number of cells of that color. We found that the different cell lines showed varying normalized clone growth speed (Fig. 2b), and that the standard deviation (SD) of clone sizes, representing clone size heterogeneity, generally correlated with the colony growth speed (Fig. 2c). Furthermore, the clonal composition kinetics changed over time, often resulting in the emergence of dominant clones, accompanied by diminishing or disappearance of others (Fig. 2d and Fig. 5Sa, c).

To further characterize the clone heterogeneity in individual colonies, we calculated their Herfindahl–Hirschman (H–H) index, used to estimate the presence of dominating subpopulations^35^, and Shannon index, used to measure the diversity of a population^36, 37^(Fig. 2e–g and Fig. S5d). We found, in all cell lines analysed, a weak trend towards increasing clone dominance and decreasing diversity over time, as shown by the positive slope of the linear fit to the average H–H indexes, and a negative slope in the case of Shannon indexes, respectively (Fig. 2e–g and Fig. S5d). There were, moreover, relatively strong correlations between clonal diversity, dominance progression and clonal intermixing (Fig. S6).

Additionally, we quantified intermixing between the different clones in each colony by calculating, for each cell, the average relative distance to its nearest neighbor of a different color (Fig. 2h), and found this distance to be relatively large in U2OS and HACAT cells, clones of which tended to be relatively concentrated in colonies as compared to RKO and LS180, with their more dispersed clones (Fig. 2a).

In aggregate, these results demonstrate the feasibility of our method in tracking various clonal properties of compact two-dimensional colonies, and reveal some differences in clonal dynamics of the selected four cancer cell lines.

### The effects of fibroblasts on clonal behavior in adherent colonies

**Figure 3:**
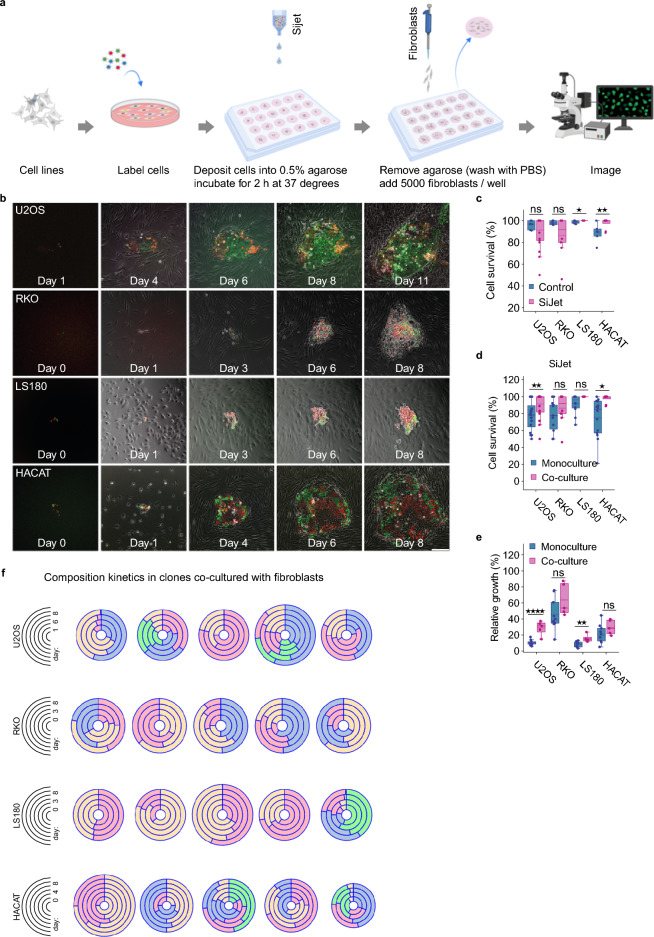
Analyzing clonal dynamics in cancer cells/fibroblasts co-cultures. (**a**) Schematic overview of the experimental pipeline, created with BioRender.com. (**b**) Representative images of selected colonies, co-cultured with normal human fibroblasts, at the indicated time points; scale bar: 200 µm. (**c**, **d**) The effect of picoliter droplet deposition on cell survival under co-culture conditions (**c**), and comparison between monoculture and co-culture (**d**), quantified as in Fig. 1b. (**e**) Relative growth of the colonies under monoculture and co-culture conditions. (**f**) Plots depicting relative growth of selected colonies and their clones over time.

To explore how the presence of other cells, often found in the neighborhood of tumor cells in vivo, affects clonal behavior, we seeded normal human fibroblasts in culture dishes with pre-deposited multiclonal cancer cell colonies (Fig. 3a, b, f). Similar to monoculture experiments, we found a limited effect of the picoliter deposition on cell survival (Fig. 3c). Interestingly, the presence of fibroblasts did appear to increase the survival of deposited cells (Fig. 3d), as compared to monoculture conditions, and the effect was statistically significant for U2OS and HACAT. The fibroblasts also significantly stimulated the growth speed in U2OS and LS180 colonies, with a similar, albeit not statistically-significant, trend observed in RKO and HACAT (Fig. 3e). Moreover, similar to monoculture experiments, clonal composition kinetics changed over time, with certain clones overwhelming the colony (Fig. 3f and Fig. S5b, c).

Next, we evaluated whether the presence of fibroblasts affects the (changes in) spatial clonal intermixing, diversity and dominance. To this end, we determined the rate of clonal dominance and diversity progression in the system by calculating the rates of change of H–H and Shannon indexes over time. The rates were obtained from the slopes of linear fits of the respective indices vs time, with a positive slope indicating a system progressing towards increased clonal dominance or diversity.

**Figure 4:**
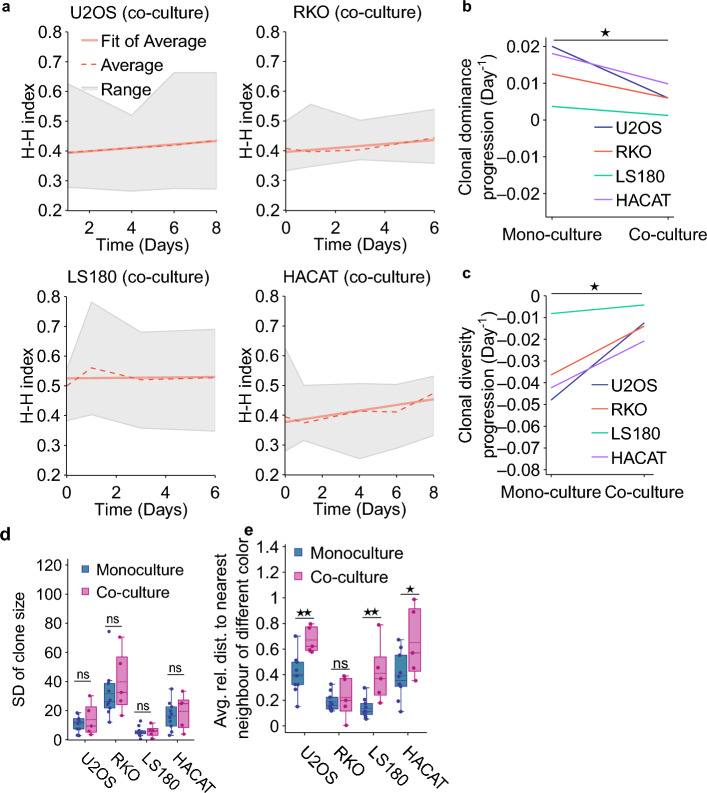
The effects of fibroblasts on clonal behavior. (**a**) Changes in the Herfindahl–Hirschman index, a measure of clonal size dominance for the indicated cell lines, under co-culture conditions. The shaded areas represent the range of the index values, dotted and continuous lines represent the mean and linear-fitted values of Herfindahl–Hirschman index at the different timepoints, respectively. (**b**) Pairwise comparison of the clonal dominance progression between mono- and co-cultures. The mean slope values of the linear fit of Herfindahl–Hirschman index values vs time data are plotted and compared per cell line. (**c**) Pairwise comparison of the clonal diversity progression, based on the Shannon index, calculated as in (**b**). (**d**) Standard deviation of clone sizes in colonies analyzed in (Fig. 3e), and comparison between monoculture and co-culture. (**e**) Clonal intermixing of indicated cell lines on day 6 and comparison between monoculture and co-culture conditions.

In general, clonal dominance progression and clonal diversity were both significantly stimulated by the presence of fibroblasts (Fig. 4a–c and Fig. S5e), nevertheless, we did not find statistically significant effects of fibroblasts on the variability in clone sizes (Fig. 4d). They did, however, significantly stimulate clonal intermixing for all cell lines except for RKO (Fig. 4e).

To further explore the impact of neighboring cells and, potentially, excreted factors on clonal behavior, we introduced either 5000 non-tagged cells or 250 μL of fibroblast-conditioned medium (FCM) into culture dishes containing pre-deposited multiclonal LS180^LeGO^ and HACAT^LeGO^ colonies. Interestingly, even though LS180^LeGO^ + HACAT or HACAT^LeGO^ + LS180 co-cultures significantly stimulated growth, the effects were comparatively less pronounced than those observed in fibroblast co-culture experiments (Fig. 3e and Fig. S7a). Moreover, there appeared to be an increasing trend in terms of clone size heterogeneity, but only the HACAT co-culture experiments demonstrated a statically significant increase (Fig. S7b). Unlike in fibroblast co-culture experiments, the addition of FCM failed to significantly increase clonal growth (Fig. S7a), aligning with the well-established notion that direct cell–cell connections play a pivotal role in cell behaviors such as proliferation, survival, and differentiation^38^. Additionally, akin to the isolated colonies and fibroblast co-culture experiments, the clonal composition kinetics of FCM and LS180^LeGO^ + HACAT or HACAT^LeGO^ + LS180 co-cultures experiments appeared to change over time, with certain clones becoming dominant, and others diminishing or disappearing (Fig. S7c–f). Furthermore, both co-cultures and FCM stimulations did appear to affect clonal diversity and dominance, yet no statistically significant differences were found (Fig. S8).

In summary, these results demonstrate the feasibility of our approach in investigating the effects of the tumor (micro)environment on the clonal dynamics in two-dimensional multiclonal colonies. They also suggest that fibroblasts may affect some aspects of clonal behavior in these simplified settings, including the cells’ migratory capacity, although further more carefully controlled experiments are required to establish the causal relationships and contribution of these effects.

### Generating and tracking multi-clonal colonies in organoid cell cultures

**Figure 5:**
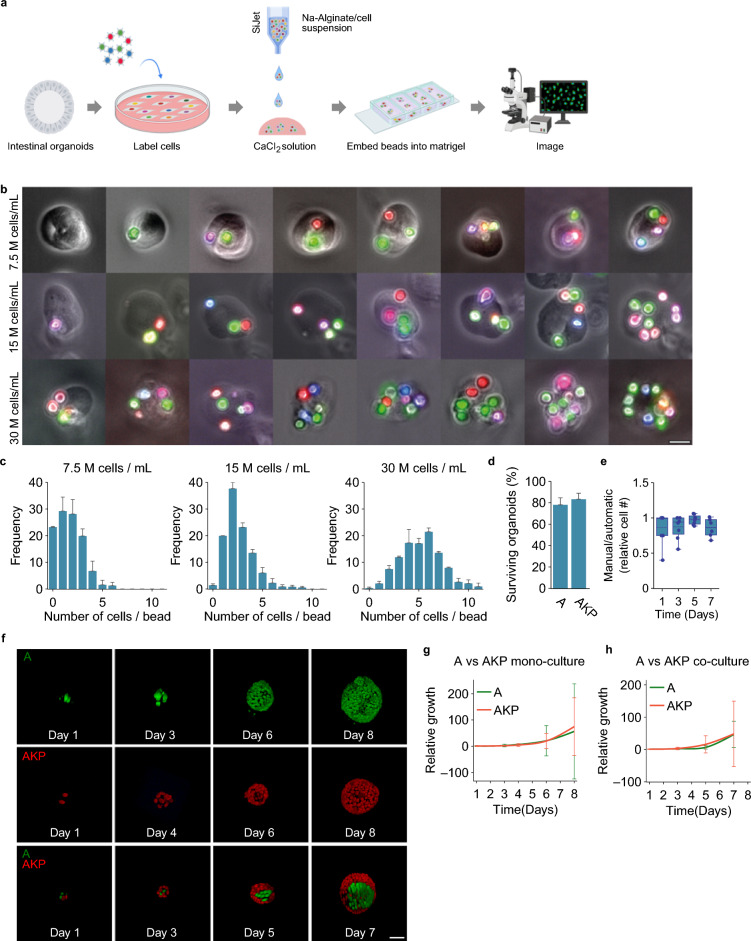
Analyzing clonal dynamics in three-dimensional multiclonal organoid cultures. (**a**) Schematic overview of the experimental pipeline, created with BioRender.com. (**b**) representative images of sodium alginate beads loaded with different numbers of cells, depending on the initial concentration of cells deposited using SiJet; scale bar: 20 µm. (**c**) Distribution of the numbers of cells encapsulated in sodium-alginate beads, depending on the initial cell concentrations. (**d**) Effect of the picoliter droplet deposition experimental procedure on the survival of organoids of the indicated genotypes. (**e**) Comparison of automated and manual cell counting to determine the number of cells. Each data point represents an individual organoid. (**f**) Representative images of selected organoids of the different genotypes at the indicated time points; scale bar: 50 µm. (**g**) Relative growth of monoclonal (i.e. containing cells of a single genotype) organoids over time. (**h**) Relative growth of bi-clonal (i.e. containing cells of both genotypes) organoids over time.

After validating our approach in two-dimensional cell cultures, we extended it to study clonal behavior in three-dimensional organoids cultured in Matrigel. To this end, we used human colon organoids, lacking either the key driver of tumor progression APC (here termed A) or a combination of APC, KRAS and p53 (here termed AKP)^39^, that were stably expressing LeGO-H2B. Dissociated cells were resuspended in sodium-alginate, and deposited, using SiJet picoliter dispenser, into CaCI_2_ solution (Fig. 5a). This produced polymerized sodium-alginate beads containing 1–10 encapsulated cells each, depending on the initial cell concentration (Fig. 5b, c). After washing, the beads were collected, mixed with matrigel, transferred to glass-bottom microscopy culture vessels and imaged in 3D at different time intervals over 7–8 days (Fig. 5f). Images are maximum-intensity projections of the 3D stacks. The organoid generation procedure exerted almost no effect on organoid viability, as compared to organoids cultured under control conditions (Fig. 5d). Images were then processed and cells were counted either by the experimenter, or by the automatic algorithm developed by Kok et al.^40^, which yielded comparable results (Fig. 5e).

To establish baseline conditions, we then analyzed the growth of A and AKP LeGO-H2B cells in monoclonal organoids, and found no significant difference in their relative growth speed (Fig. 5g). Similar results were obtained when analyzing bi-clonal organoids comprising A and AKP cells (Fig. 5h).

In conclusion, these results demonstrate that the picoliter deposition method is suitable for analyzing clonal dynamics in three-dimensional, multi-clonal mouse colon organoids.

## Discussion

In this brief report we present a set of straightforward methods for generating small, spatially isolated, multiclonal, fluorescently-barcoded colonies of adherent and three-dimensional cell cultures, and for analyzing a number of key properties of clonal dynamics within these colonies, over time, using fluorescence microscopy. We also show results of proof-of-concept experiments comparing these properties among a number of cancer cell lines, as well as under mono-, and fibroblast co-culture conditions.

Among key limitations of the proposed method is the relatively short time during which the colonies can be followed. In the case of adherent cultures, this limitation mainly stems from the fact that after reaching the size of a few hundred cells, the cores in colonies of most cell lines become relatively dense and cells start growing on top of each other, often in multiple layers, which makes the imaging and analysis challenging. Although we do not present the relevant data in this study, we did obtain promising initial results with long-term imaging (1–1.5 months) when each individual colony was cultured under a glass 25 mm coverslip, which limited the upward growth of colony centers. We are currently further exploring this modification. In the case of three-dimensional cell cultures, after ~ 1 week the organoids generally become too large for imaging along the optical axis using standard wide-field and confocal fluorescence microscopy techniques. We decided, therefore, to stop our experiments after approximately 1–1.5 weeks. Such large structures may be, however, suitable for long-term imaging using techniques such as light-sheet microscopy^41^.

Another challenge is related to reliable detection and assignment of clonal progeny in colonies composed of clones with similar colors, when using cells that can, in principle, express any and all fluorophores at different ratios, resulting in a wide gamut of colors. To mitigate this issue, when studying 3D cell cultures, we used cells that were transduced with predefined combinations of the red, green and blue fluorescent tags, and we based color assignment on the presence, rather than on the intensity of fluorescence of each tag. This limited the number of possible color combinations to seven, which was sufficient in most cases as our colonies contain a few clones on average. The range of detectable colors could be extended by adding additional fluorophores with different spectral properties.

Among the key advantages of our method is the simplicity of colony generation and deposition using the SiJet picoliter dispenser. We have not explored other small-volume dispensers, but they should be compatible if droplets of picoliter-range can be generated. The process can also be fully automated, such that a predetermined number of cell-containing droplets are dispensed at predetermined locations into one-well or multi-well plates for higher throughput. In our case, this was achieved by connecting the nanodispenser to a microscope stand with an automatic stage controlled by a custom software; commercial, fully automated dispensing stations are also available, albeit at a significantly higher cost. One consequence of the adapted approach, however, is that it is not feasible to predetermine or predict the exact composition of each colony, as fluorescently tagged cells are randomly distributed into the individual droplets.

In our proof-of-concept experiments, we explored some basic clonal parameters of various cancer cell lines, mostly focusing on adherent cultures. We found a relatively large heterogeneity of clone growth rates in RKO and HACAT cells, as compared to U2OS and LS180. Some gene expression or proteomics data generated from single cells are available for some of these cell lines, and generally reveal large intra-cell-line heterogeneity (e.g. RKO, U2OS)^42, 43^, but, to our knowledge, a direct comparison of genomic or (epi)genetic heterogeneity in these cell lines has not been performed in a single study. It is, therefore, challenging to pinpoint the mechanisms driving the clone growth heterogeneity we observed. Interestingly, the clone growth heterogeneity correlated with increased colony growth rates, which could, in principle, amplify initially small differences. More experiments with analyses at timepoints selected to match the number of cell divisions for each cell line (rather than identical time-points for all cell lines) could reveal to what extent the growth rate determines the clone size heterogeneity.

The clone size heterogeneity did not clearly correlate with clonal diversity or dominance progression (Fig. 2e–g), nor with clonal intermixing (Fig. 2h). There appears to be, however, an inverse correlation between clonal diversity and dominance progression and, interestingly, between these and clonal intermixing (Fig. S6). As clonal intermixing is the result of the redistribution of cells among an unrelated clone in a given colony, and thus (likely) a product of cell migration and cell–cell contact strength, while clonal dominance and diversity are related to clone sizes alone, the mechanism underpinning this correlation is difficult to envision. Experiments with more cell lines will be required, however, to validate these results.

Addition of non-transformed fibroblasts generally stimulated colony growth rates, in line with published data^44, 45^ (Fig. 3e). Interestingly, fibroblasts increased the clonal intermixing for two out of four cell lines and, to a relatively small degree, the clonal diversity progression, and reduced the clonal dominance progression (Fig. 4). Results of some studies have suggested that cancer associated fibroblasts (CAFs) can contribute to tumor heterogeneity by promoting the development of subpopulations of cancer cells with different genetic and epigenetic profiles. Additionally, CAFs can produce and release a variety of growth factors and extracellular matrix components that can promote the development of different cell types within the tumor, including cancer stem cells, further contributing to its heterogeneity^44, 46–51^. It will be interesting to explore whether and how the heterogeneity of simple parameters measured here, such as clone growth rate or clonal dominance, reflect differences in these complex underlying mechanisms.

The results of experiments involving colon organoids of different genotypes are somewhat surprising; whereas we did not measure significant differences between these genotypes in cell proliferation, regardless of whether different genotypes were analyzed in mono- or co-culture conditions, dysfunctions in KRAS and p53 are known to stimulate cell proliferation^52–55^. More experiments with additional genotypes relevant in colorectal cancer progression are ongoing to investigate this further.

In conclusion, this study presents a new method for studying clonal dynamics in two- and three-dimensional in vitro cancer cell cultures. By combining picoliter droplet deposition and fluorescent markers of cell lineage, we were able to track the growth and proliferation of individual cancer cell clones in multiclonal colonies over time. Our results provide some initial correlations between clonal growth parameters that can help to understand the mechanisms underlying clonal evolution in cancer. Further studies are needed, however, to validate our findings using different methods, with single-cell omics techniques especially suitable to link heterogeneity to simple parameters describing clonal growth dynamics^56^. Our method could also prove suitable for studying clonal dynamics in vivo after injecting pre-generated multiclonal colonies into mice*,* and we have initiated a series of pilot experiments to test such an application. The new method we propose here could also provide a powerful tool for understanding the complex interactions between cancer cells and the microenvironment that are important for cancer progression, and may ultimately help to identify new targets for the development of more effective cancer therapies.

## Materials and methods

### Cell culture

RKO and LS180 cell lines (Sanger Institute) were cultured in DMEM/F12 (Gibco) and U2OS, HACAT, human fibroblast cell lines (ATCC) were cultured in Dulbecco’s modified Eagle medium (DMEM) (Gibco). Both medium formulations were supplemented with 10% fetal bovine serum (Gibco) and 1% Penicillin–Streptomycin (Gibco) and l-glutamine (Gibco). Cell lines were incubated at 37 °C and 5.0% CO_2_.

### Organoid culture

Human intestinal organoids (kindly provided by Jarno Drost) were previously described^39^ and cultured in a basal organoid medium containing advanced DMEM/F12 medium including 100× N-2 and 50× B-27 supplements, 100× GlutaMAX, and 100× antibiotic/antimycotic (all from Gibco). R-spondin (conditioned medium), Noggin (conditioned medium), EGF (Peprotech), 1 mM *N*-acetyl-l-cysteine (Sigma-Aldrich), nicotinamide (Sigma-Aldrich), TGF-β type I receptor inhibitor A83-01 (Tocris), P38 inhibitor SB202190 (Sigma-Aldrich), gastrin (Sigma-Aldrich), and gentamycin (Lonza) were added to the basal medium.

### Generation of nuclear derivatives of lentiviral gene ontology (LeGO) markers

Nuclear localization signal (NLS) of human c-Myc proto-oncogene (3′ GGACGACGCTTCTCCCAGTTTAACCTG 5′)^57, 58^ or human histone H2B gene sequence (3′ GGTCTCGGTCGCTTCAGACGAGGGCGGGGCTTTTTCCCGAGGTTCTTCCGCCACTGATTCCGCGTCTTCTTTCCGCCGTTCTTCGCGTTCGCGTCGGCGTTCCTCTCGATAAGGTAGATACACATGTTCCAAGACTTCGTCCAGGTGGGACTGTGGCCGTAAAGCAGGTTCCGGTACCCGTAGTACTTAAGCAAACACTTGCTGTAAAAGCTCGCGTAGCGTCCACTCCGAAGGGCGGACCGCGTAATGTTGTTCGCGAGCTGGTAGTGGAGGTCCCTCTAGGTCTGCCGGCACGCGGACGACGACGGACCCCTCAACCGGTTCGTGCGGCACAGGCTCCCATGATTCCGGTAGTGGTTCATGTGGTCGCGATTC 5′) were inserted into LeGO-C2 (27339), LeGO-V2 (27340), and LeGO-Cer2 (27338) (Addgene) vectors for nuclear visualisation, using standard DNA cloning protocols. Cells were transduced with lentiviral gene ontology (LeGO) markers in accordance with previously published works by Weber et al.^25^ and Heijen et al.^18^.

#### Transduction of cell lines

Briefly, 50,000 cells were seeded in a single well of a 12-well plate in 1 mL culture medium and incubated at 37 °C and 5.0% CO_2_ for ~ 24 h, at ~ 70% confluency. 50 μL of concentrated lentivirus encapsulating LeGO-NLS DNA, generated using standard protocols, was added into 1 mL culture medium in the presence of 8 μg/mL polybrene (Sigma-Aldrich) and incubated overnight at 37 °C and 5.0% CO_2_.

#### Transduction of organoids

Organoid cultures exponentially growing in 50 μL Matrigel (Corning) domes were used for transduction. Medium was removed and organoids were dissociated with 500 μL ice-cold Cell Recovery Solution (Corning) and collected into 15 mL tubes and vigorously pipetted to break large organoids into smaller clumps. These were then incubated on ice for 30 min to dissolve the Matrigel. Subsequently, organoids were centrifuged for 4 min at 225 g and then supernatant was removed. 8 μg/mL Polybrene and 10 μM Y-27632 dihydrochloride (ROCK inhibitor) were added to 250 μL human organoid culture medium, mixed with the organoids and transferred into a 48-well plate. 40 μL of concentrated lentivirus containing LeGO-H2B DNA was then added into each well. Plates were centrifuged at 507 g for 30 min and incubated overnight at  C and 5.0% CO_2_. Next day, organoids were collected into 15 mL tubes and centrifuged for 4 min at 225 g*.* Supernatant was decanted and organoids were washed with 1 mL of PBS and centrifuged for 4 min at 225 g. The washing was repeated three times. Transduced organoids were resuspended in 100 μL of Matrigel, divided over two wells of pre-warmed 24 well plates. Plates were then turned upside-down in the incubator for 5–10 min until Matrigel polymerized. Finally, 500 μL human organoid medium supplemented with 10 μM ROCK inhibitor (Y-27632) was added to each well. SH800 Sony cell sorter was used to select the stably transduced cells 3–5 days later.

### Generation of multi-clonal colonies

#### Adherent cell cultures

0.5% low-melting agarose (Bio Rad) solution was first prepared in DMEM medium by heating it to ~ 60 °C. After cooling the solution to ~ 37 °C, it was supplemented with FBS to reach the end concentration of 10%. The solution was then filtered through a 0.22 μm filter (Millex) and stored at 37–50 °C for up to a few days. To generate colonies, trypsinized cells were concentrated to 10–15 million cells/mL in > 100 μL of culture medium and filtered through a sterile 50 μm filter (CTSV). 50 μL of the cell suspension was then loaded into the reservoir of the SiJet Picodispencer P9 (Biofluidix) with the following settings: Stroke (S) = 100%, str. velocity = 125 μm/ms Frequency = 6 Hz. Droplets containing ~ 1–10 cells were deposited into wells of a 24-well plate pre-filled with 100 μL of 0.5% low-melting-point agarose solution to reduce cell movement during transportation to the incubator and prevent droplet evaporation. The plates were stored for 10 min at room temperature to allow agarose polymerization before transfer to incubators and two-hour incubation to allow attachment of cells to the bottom of the culture plates. Agarose was then gently replaced with cell culture medium. Plates were incubated at 37 °C and 5.0% CO_2_ and imaged over time. In cell-fibroblast co-culture experiments, 5000 fibroblasts were additionally seeded into each well after removing the agarose. In cell–cell co-culture experiments 5000 non-tagged HACAT and 5000 non-tagged LS180 were additionally seeded, respectively, on pre-deposited multiclonal LS180^LeGO^ and HACAT^LeGO^ colonies. For the control group 5000 non-transformed the same cells were seeded on pre-deposited multiclonal colonies.

#### Three-dimensional cell cultures

Before start, 0.125%, pH 7.4 sodium-alginate solution was prepared with a 13 mM HEPES buffer (Sigma) containing 0,84% NaCl (Merck). The solution was filtered through a 0.22 μm filter (Millex). 100 mM CaCI_2_ solution was prepared in the organoid culture medium and filtered through a 0.22 μm filter (Millex).

At least 6 wells of healthy organoid cultures grown in 50 μL Matrigel domes in 24 well-plates were needed for generation of multi-clonal organoids. Medium was removed and organoids were dissociated with 250 μL ice-cold Cell Recovery Solution (Corning), for each well, collected into 15 mL tubes and vigorously pipetted to dissociate clumps of organoids. Cells were incubated in ice for 30 min to allow Matrigel dissolution. Subsequently, organoids were centrifuged for 4 min at 225 g and the supernatant was discarded. 400 μL TrypLE Express (Invitrogen) was added to the cell pellet and mixed well. Cells were then incubated at 37 °C and pipetted every 2–3 min until obtaining single-cell suspension, which was then filtered through a sterile 50 μm filter (CTSV) and centrifuged at 225 g for 4 min.

After removing the supernatant, cells were mixed well with 500 μL organoid culture medium, counted, and resuspended in sodium-alginate volume to obtain required cell concentration. 50 μL of the suspension was then loaded into SiJet P9 Picodispencer (Biofluidix) with the following settings: Stroke (S) = 100%, Str. Velocity (SV) = 175 μm/ms, Frequency = 5 Hz. Subsequently, 8000 of droplets were deposited into 100 mM CaCI_2_ solution in a small petri dish for encapsulation of the cells into the sodium-alginate beads. After 5 min of incubation at room temperature, suspension was transferred into a 15 mL tube and centrifuged for 4 min at 225 g. Supernatant was discarded, the pellet was washed with 2 mL FBS, centrifuged for 4 min at 225 g and supernatant was discarded. Beads were resuspended in 100 μL of Matrigel and plated in 2 wells of a 4-well plate optical-bottom dish (Ibidi). Dishes were incubated on ice for 6 min, then for 12 min upside-down in the incubator at 37 °C and 5.0% CO_2_. 500 μL human organoid medium supplemented with 10 μM ROCK inhibitor (Y-27632) was then added to each well. Organoids were imaged starting on the subsequent day.

### Imaging

Two-dimensional cultures were imaged using a Leica DMI8 microscope using 10× objective. Chanel filter Da/FI/TX was used which uses EX:394-412;483-501;562-588, DC:535;505;595 and EM: 441-471;512-548;600-660 for mCherry, mVenus and mCerulean.

For quantifying cell survival after picoliter deposition, cells were imaged for four days at 30 min intervals, with a Leica DMi8 microscope in phase-contrast mode at 10× magnification, 37 °C and 5.0% CO_2_. Imaging was initiated four hours after the generation of colonies. 3D organoids were imaged with the Leica SP8X confocal microscope using a 20× objective. 515 nm and 587 nm wavelengths for excitation, 527 and 610 wavelengths for emission of mVenus and mCherry were used, respectively.

### Image analysis

The analysis and quantification of images was done manually and semi-automatically. For manual quantification, montages of fluorescence images from subsequent time points were generated to facilitate the color matching and counting. To enable semi-automatic cell counting in images of adherent cultures, a Matlab script called CI Imaging was custom-written. Starting from the raw fluorescence image (Fig. S2a), the script first filters the image, removing background noise (Fig. S2b). The individual cell nuclei are then segmented and labeled (Fig. S2c). The average color hue value for each nucleus is then calculated and enhanced (Fig. S2d). To automatically determine cells belonging to each color clone, a histogram is generated by plotting the number of pixels of a given hue value against the hue, excluding the background pixels (Fig. S2e). Clones are then identified by automatic peak detection in the hue histogram, and clone identity is then assigned to nuclei based on their median hue value.

The analysis and quantification of organoids was done manually and updated version of the OrganoidTracker algorithm developed by Kok et al.^40^.

### Data analysis

To analyze clone intermixing, the relative nearest-neighbor distance (RNND) of clones in a colony was determined. First, each colony was deconstructed to X and Y coordinates of cell nuclei of each clone. Then, the distances between each nucleus in a given clone to its nearest same-clone neighbor (d0), and to its nearest neighbor in each of the other clones (d1, d2, …, dn) were determined and averaged per clone and then per colony.

Shannon^37^ and Hirschman-Herfindahl^35^ indices were used to calculate the clonal diversity and dominance respectively. The slopes of the linear fit of the indices against time were used to measure the rates of diversity and dominance progression in time. A negative diversity progression rate indicates that a clonal population becomes more homogenous (with fewer different clones) and vice versa. On the other hand, a negative dominance progression rate signals the emergence of equal competition or growth of clonal populations in time.

### Statistical analysis

Statistical analyses were performed using GraphPad Prism 9. Significance was tested with unpaired or paired (Fig. 4b, c and Fig. S2g) Student’s t-tests. Ns = not significant, *p < 0.05, **p < 0.01, ***p < 0.001, ****p < 0.0001.

### Supplementary Information

Supplementary Figures.

## Data Availability

The datasets generated and/or analysed during the current study are available in the FiglinQ repository, [https://create.figlinq.com/~s.baglamis/221/].

## References

[1] Zheng Z (2020). Intratumor heterogeneity: A new perspective on colorectal cancer research. Cancer Med..

[2] Burrell RA, McGranahan N, Bartek J, Swanton C (2013). The causes and consequences of genetic heterogeneity in cancer evolution. Nature.

[3] Oh BY (2019). Intratumor heterogeneity inferred from targeted deep sequencing as a prognostic indicator. Sci. Rep..

[4] Schmidt F, Efferth T (2016). Tumor heterogeneity, single-cell sequencing, and drug resistance. Pharmaceuticals.

[5] Marusyk A, Polyak K (2010). Tumor heterogeneity: Causes and consequences. Biochim. Biophys. Acta Rev. Cancer.

[6] Merlo LMF, Pepper JW, Reid BJ, Maley CC (2006). Cancer as an evolutionary and ecological process. Nat. Rev. Cancer.

[7] Farrokhian N (2022). Measuring competitive exclusion in non-small cell lung cancer. Sci. Adv..

[8] Nishikawa S, Takamatsu A, Ohsawa S, Igaki T (2016). Mathematical model for cell competition: Predator–prey interactions at the interface between two groups of cells in monolayer tissue. J. Theor. Biol..

[9] van Neerven SM (2021). Apc-mutant cells act as supercompetitors in intestinal tumour initiation. Nature.

[10] Krotenberg Garcia A (2021). Active elimination of intestinal cells drives oncogenic growth in organoids. Cell Rep..

[11] Vermeulen L (2013). Defining stem cell dynamics in models of intestinal tumor initiation. Science.

[12] Boone PG (2019). A cancer rainbow mouse for visualizing the functional genomics of oncogenic clonal expansion. Nat. Commun..

[13] Baker AM (2014). Quantification of crypt and stem cell evolution in the normal and neoplastic human colon. Cell Rep..

[14] Van Leeuwen IMM (2009). An integrative computational model for intestinal tissue renewal. Cell Prolif..

[15] Snippert HJ, Schepers AG, Van Es JH, Simons BD, Clevers H (2014). Biased competition between Lgr5 intestinal stem cells driven by oncogenic mutation induces clonal expansion. EMBO Rep..

[16] Enquist IB (2014). Lymph node-independent liver metastasis in a model of metastatic colorectal cancer. Nat. Commun..

[17] de-Sousa-e-Melo F (2020). Modeling colorectal cancer progression through orthotopic implantation of organoids. Methods Mol. Biol..

[18] Van Der Heijden M (2019). Spatiotemporal regulation of clonogenicity in colorectal cancer xenografts. Proc. Natl. Acad. Sci. U. S. A..

[19] Alexandrov LB (2013). Signatures of mutational processes in human cancer. Nature.

[20] Porter SN, Baker LC, Mittelman D, Porteus MH (2014). Lentiviral and targeted cellular barcoding reveals ongoing clonal dynamics of cell lines in vitro and in vivo. Genome Biol..

[21] Sankaran VG, Weissman JS, Zon LI (2022). Cellular barcoding to decipher clonal dynamics in disease. Science.

[22] Serrano A, Berthelet J, Naik SH, Merino D (2022). Mastering the use of cellular barcoding to explore cancer heterogeneity. Nat. Rev. Cancer.

[23] Bhang H-EC (2015). Studying clonal dynamics in response to cancer therapy using high-complexity barcoding. Nat. Med..

[24] Acar A (2020). Exploiting evolutionary steering to induce collateral drug sensitivity in cancer. Nat. Commun..

[25] Weber K, Thomaschewski M, Benten D, Fehse B (2012). RGB marking with lentiviral vectors for multicolor clonal cell tracking. Nat. Protoc..

[26] van Neerven SM, Ramadan R, van Driel MS, Huels DJ, Vermeulen L (2022). Intestinal organoid co-culture protocol to study cell competition in vitro. STAR Protoc..

[27] Mohme M (2017). Optical barcoding for single-clone tracking to study tumor heterogeneity. Mol. Ther..

[28] Shembrey C (2022). Longitudinal monitoring of intra-tumoural heterogeneity using optical barcoding of patient-derived colorectal tumour models. Cancers Basel.

[29] Weber K, Bartsch U, Stocking C, Fehse B (2008). A multicolor panel of novel lentiviral “Gene Ontology” (LeGO) vectors for functional gene analysis. Mol. Ther..

[30] Weber K (2011). RGB marking facilitates multicolor clonal cell tracking. Nat. Med..

[31] Brenière-Letuffe D (2018). Clonal dynamics studied in cultured induced pluripotent stem cells reveal major growth imbalances within a few weeks. Stem Cell Res. Ther..

[32] Bolck HA (2021). Tracing clonal dynamics reveals that two- and three-dimensional patient-derived cell models capture tumor heterogeneity of clear cell renal cell carcinoma. Eur. Urol. Focus.

[33] Kanda T, Sullivan KF, Wahl GM (1998). Histone–GFP fusion protein enables sensitive analysis of chromosome dynamics in living mammalian cells. Curr. Biol..

[34] Hadjantonakis AK, Papaioannou VE (2004). Dynamic in vivo imaging and cell tracking using a histone fluorescent protein fusion in mice. BMC Biotechnol..

[35] Rhoades SA (1993). The Herfindahl-Hirschman index. Federal Reserve Bull..

[36] Park SY, Gönen M, Kim HJ, Michor F, Polyak K (2010). Cellular and genetic diversity in the progression of in situ human breast carcinomas to an invasive phenotype. J. Clin. Invest..

[37] Colwell RK (2014). Biodiversity: Concepts, patterns, and measurement. Princeton Guide Ecol..

[38] Jiao Q (2017). Cell-cell connection enhances proliferation and neuronal differentiation of rat embryonic neural stem/progenitor cells. Front. Cell Neurosci..

[39] Drost J (2015). Sequential cancer mutations in cultured human intestinal stem cells. Nature.

[40] Kok RNU (2020). OrganoidTracker: Efficient cell tracking using machine learning and manual error correction. PLoS ONE.

[41] Voie AH, Burns DH, Spelman FA (1993). Orthogonal-plane fluorescence optical sectioning: Three-dimensional imaging of macroscopic biological specimens. J. Microsc..

[42] Mund A (2022). Deep visual proteomics defines single-cell identity and heterogeneity. Nat. Biotechnol..

[43] Park SR (2020). Single-cell transcriptome analysis of colon cancer cell response to 5-fluorouracil-induced DNA damage. Cell Rep..

[44] Lenos KJ (2018). Stem cell functionality is microenvironmentally defined during tumour expansion and therapy response in colon cancer. Nat. Cell Biol..

[45] Vermeulen L (2010). Wnt activity defines colon cancer stem cells and is regulated by the microenvironment. Nat. Cell Biol..

[46] Su S (2018). CD10+GPR77+ cancer-associated fibroblasts promote cancer formation and chemoresistance by sustaining cancer stemness. Cell.

[47] Samoszuk M, Tan J, Chorn G (2005). Clonogenic growth of human breast cancer cells co-cultured in direct contact with serum-activated fibroblasts. Breast Cancer Res..

[48] Shen Z (2016). Cancer-associated fibroblasts promote cancer cell growth through a miR-7-RASSF2-PAR-4 axis in the tumor microenvironment. Oncotarget.

[49] Shiga K (2015). Cancer-associated fibroblasts: Their characteristics and their roles in tumor growth. Cancers.

[50] Yamamura Y (2015). Akt-girdin signaling in cancer-associated fibroblasts contributes to tumor progression. Cancer Res..

[51] Suzuki J, Tsuboi M, Ishii G (2022). Cancer-associated fibroblasts and the tumor microenvironment in non-small cell lung cancer. Expert Rev. Anticancer Therapy.

[52] Sameer AS (2013). Colorectal cancer: Molecular mutations and polymorphisms. Front. Oncol..

[53] Barbacid, M. ras genes. Annu Rev Biochem **56**, 779–827 (1987). 10.1146/annurev.bi.56.070187.004023 3304147

[54] Khosravi-Far R, Der CJ (1994). The Ras signal transduction pathway. Cancer Metastasis Rev..

[55] Harris CC, Hollstein M (1993). Clinical implications of the p53 tumor-suppressor gene. N. Engl. J. Med..

[56] Nam AS, Chaligne R, Landau DA (2020). Integrating genetic and non-genetic determinants of cancer evolution by single-cell multi-omics. Nat. Rev. Genet..

[57] Dang CV, Lee WMF (1988). Identification of the human c-myc protein nuclear translocation signal. Mol. Cell Biol..

[58] Baglamis S (2023). A novel high-throughput framework to quantify spatio-temporal tumor clonal dynamics. Int. Conf. Comput. Sci..

