# Supplementary material for: Using picoliter droplet deposition to track clonal competition in adherent and organoid cancer cell cultures
Source: Sci Rep. 2023 Nov 1;13:18832. doi: 10.1038/s41598-023-42849-w (PMC10620187; doi:10.1038/s41598-023-42849-w)
Supplement: Supplementary file 1 — Supplementary Figures. [file 41598_2023_42849_MOESM1_ESM.pdf]

Using picoliter droplet deposition to track clonal competition in adherent and organoid cancer cell cultures

Selami Baglamis[1,2,3,4], Vivek M Sheraton[1,2,3,4,5]†, Debora Meijer[2,6]†, Haibin Qian[2,6], Ron A. Hoebe[2,6], Max A. Betjes[7], Sander Tans[7], Jeroen van Zon[7], Louis Vermeulen[1,2,3,4]*, Przemek M. Krawczyk[2,6]*.

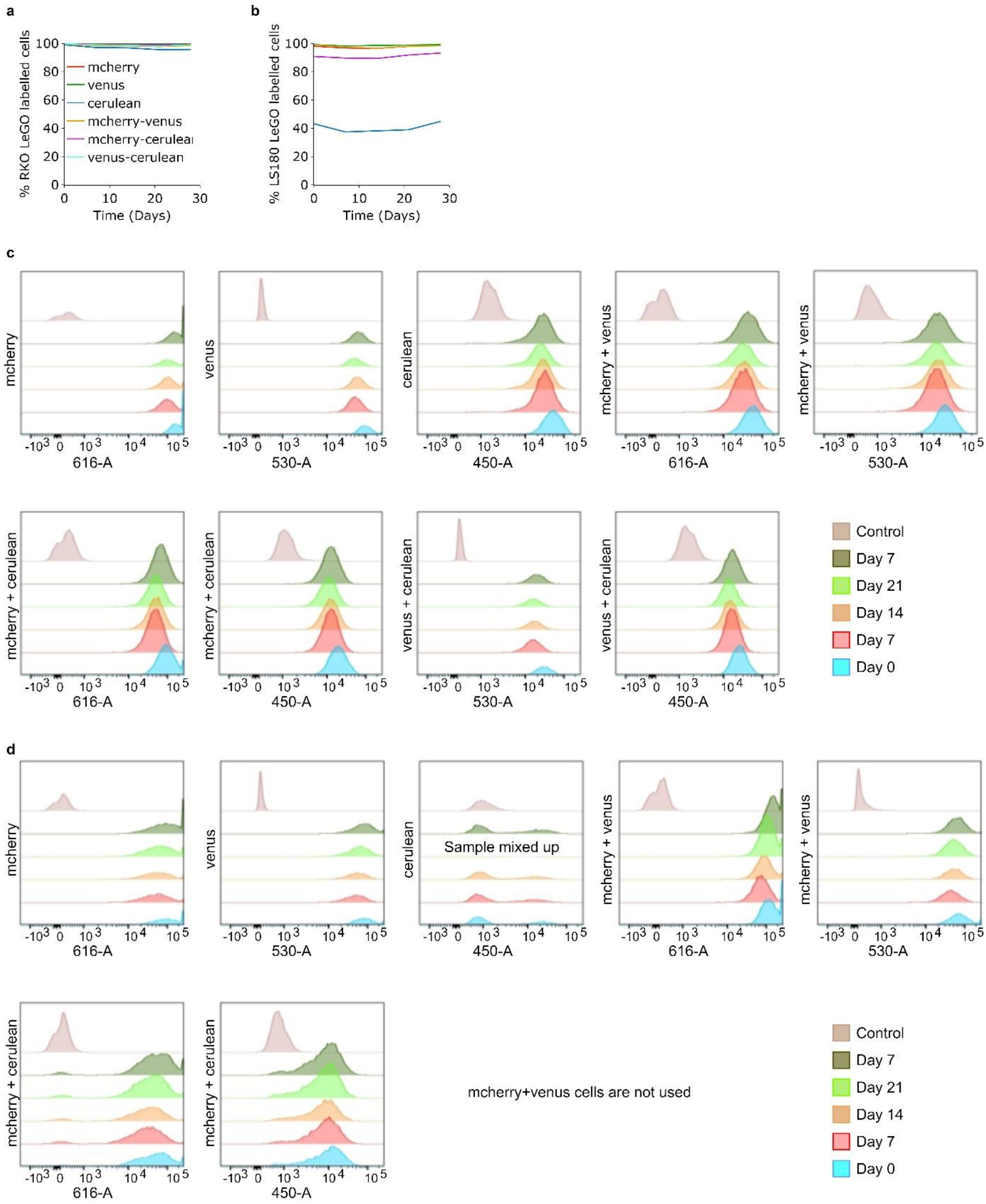

Supplementary figure 1. Stability of LeGO fluorescent optical tags in cell populations. The stability of LeGO fluorescent optic tags was assessed by FACS over a span of 28 days. (**a,b**) Changes in percentage of RKO and LS180 cell lines. (**c,d**) Raw intensity of RKO and LS180 cell lines over the time, respectively.

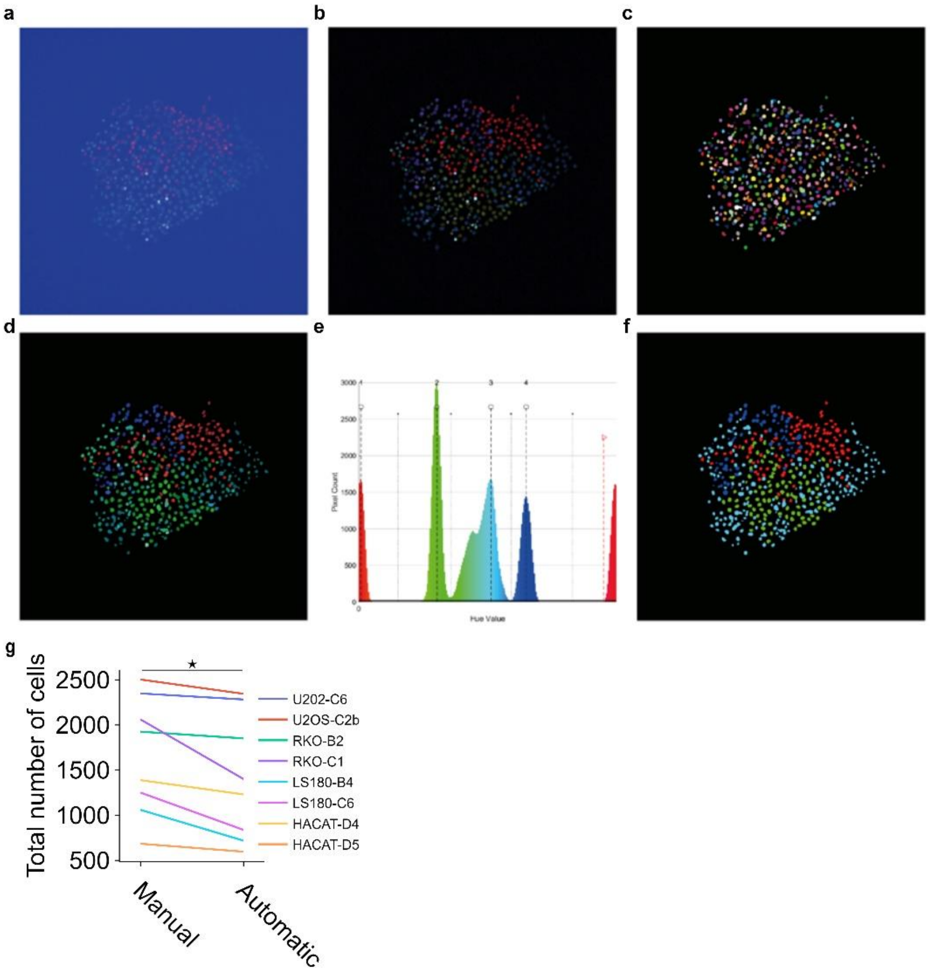

Supplementary figure 2. Image analysis pipeline. The images represent subsequent steps in the image processing pipeline. (**a**) Original image. (**b**) Background-corrected image. (**c**) Cell nuclei masks. (**d**) Cell nuclei masks with Initial color assignment based on fluorescence. (**e**) Circular color histogram with automatically detected peaks. (**f**) Cell nuclei masks with applied colors based on circular histogram peak finding. (**g**) Comparison of the results of automated and manual nuclei detection and color assignment.

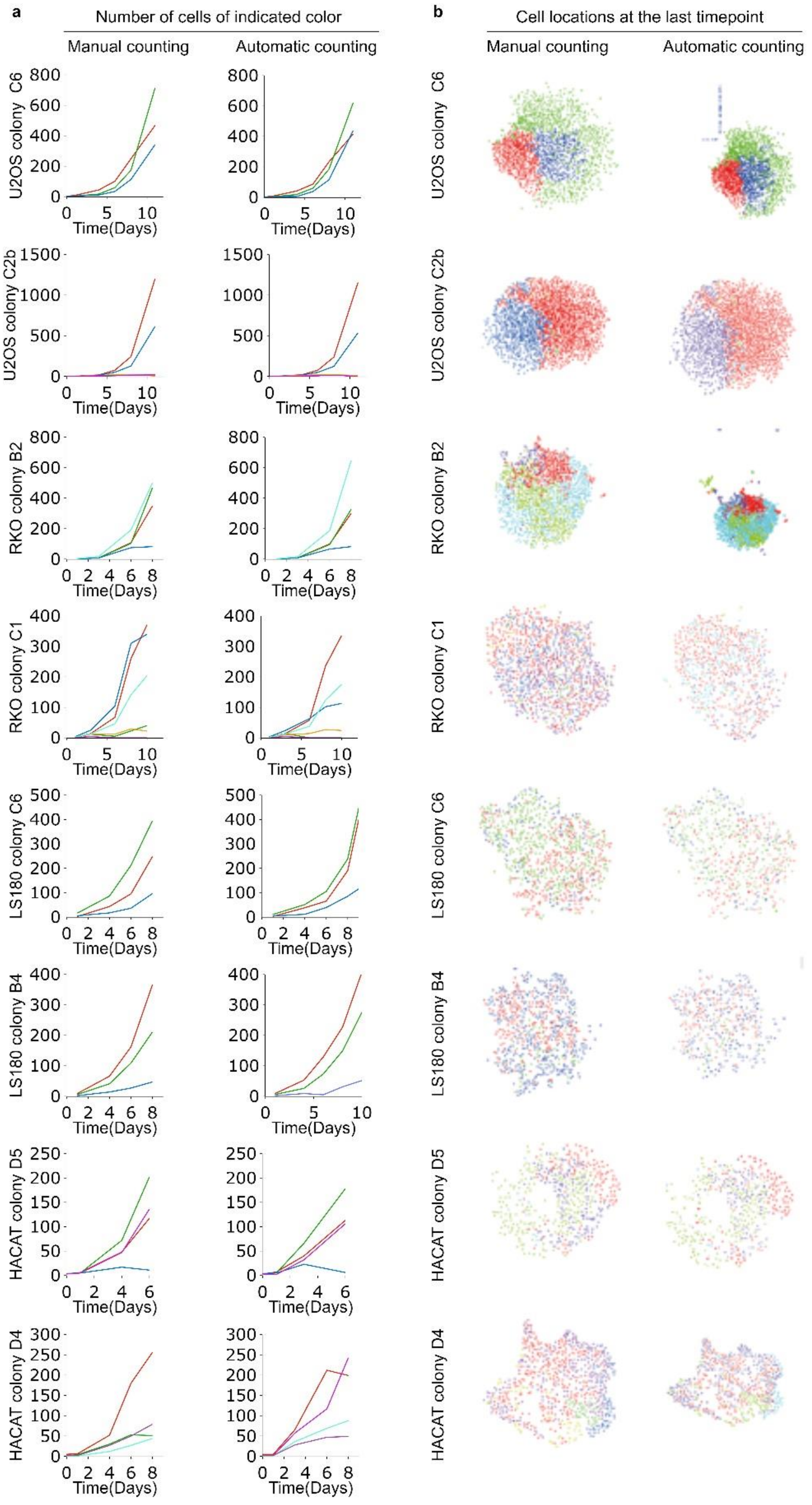

Supplementary figure 3. Detailed comparison of automated and manual finding of cells in two-dimensional multi clonal colonies. (**a**,**b**) Quantification (**a**) and visual representation (**b**) of the differences between manual and automatic cell detection for two randomly selected, two-dimensional colonies of each cell line.

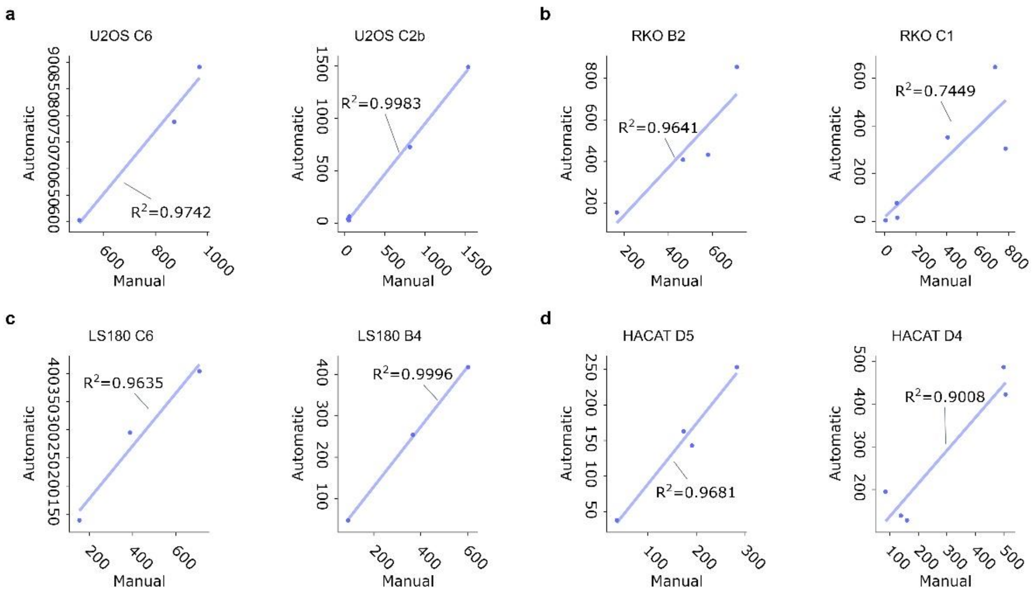

Supplementary figure 4. Correlation between results of manual and automatic cell counting. The data from **Fig. S3a** was utilized to compute the correlation between manual and automatic counting for each respective cell line. (**a**) U2OS. (**b**) RKO. (**c**) LS180. (**d**) HACAT.

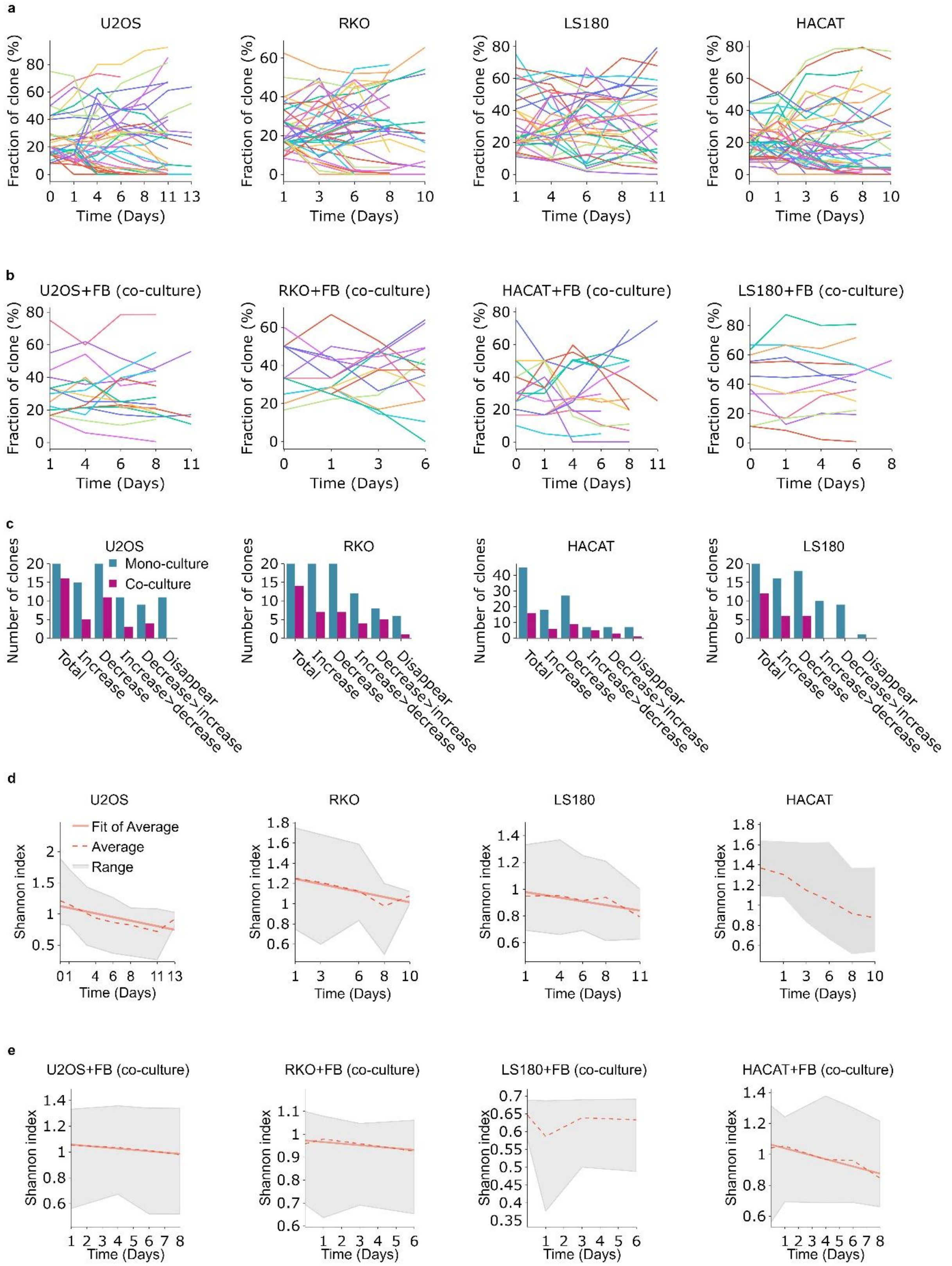

Supplementary figure 5. Analyzing clonal kinetics in mono- and co-cultures. (**a,c**) Changes in clonal composition kinetics over time for monoculture and cell-fibroblast co-culture (adapted from **Fig. 2d** and **Fig. 3f**). Each graph displays every clone examined in the specified cell line. On these graphs, every

line illustrates the percentage that a particular clone contributes to its own colony. (**c**) Alteration in clonal number kinetics over time: increase, decrease, increase followed by decrease, decrease followed by increase, disappearance. Adapted from **Fig. S5a, b**. (**d,e**) Changes in the Shannon index, a measure of clonal diversity, for the indicated cell lines, under monoculture and fibroblast co-culture conditions. The shaded areas represent the range of the index values, dotted and continuous lines show the mean and linear-fitted values of Shannon index at the different timepoints, respectively.

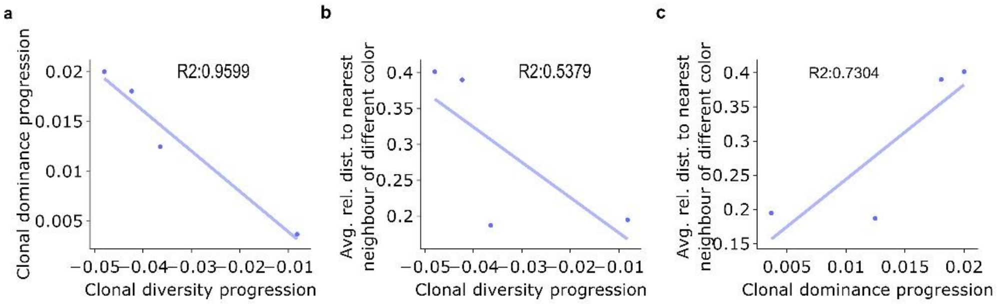

Supplementary figure 6. Pairwise correlations between clonal diversity, dominance progression and clonal intermixing. Related to figure 2.

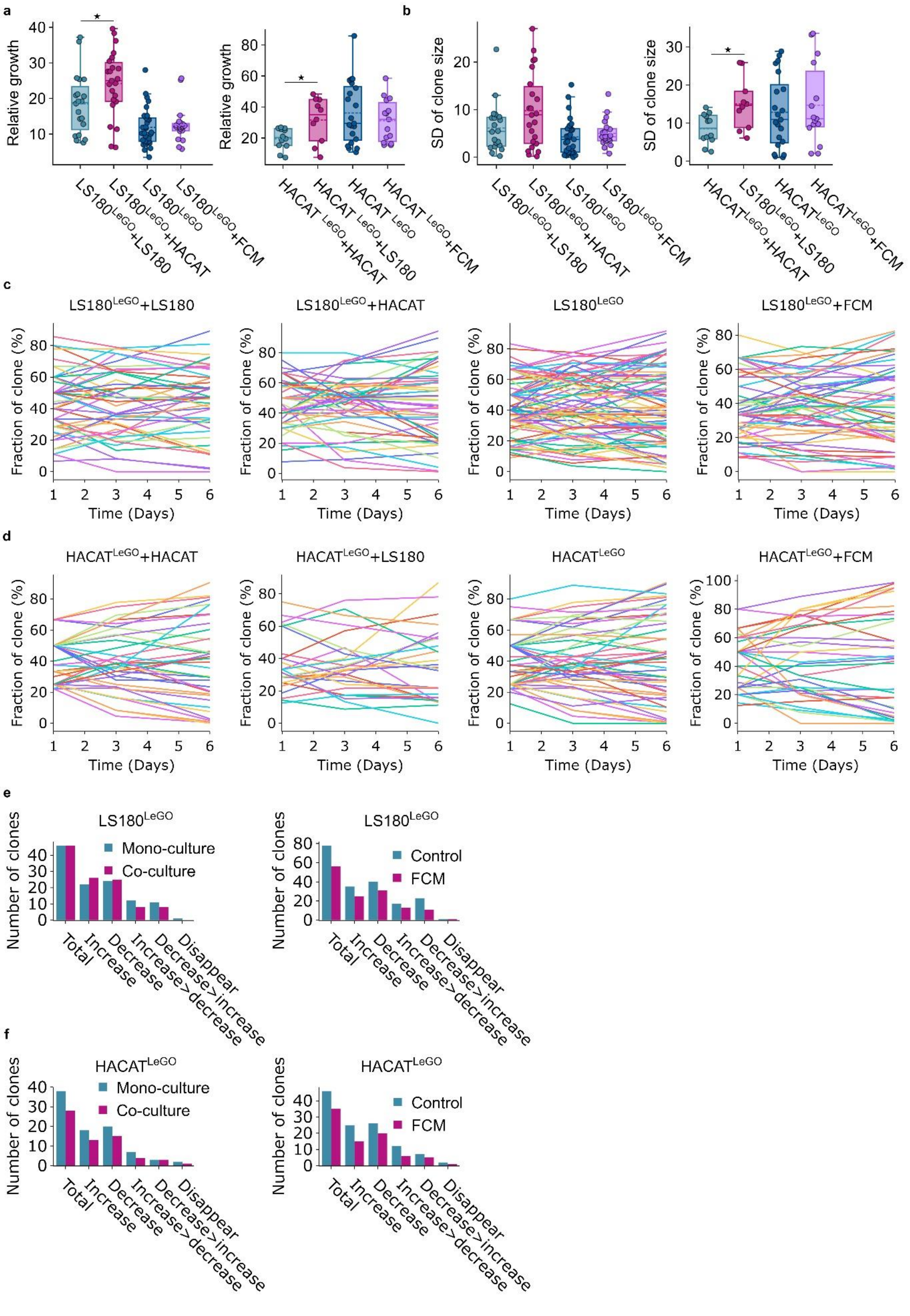

Supplementary figure 7. The effect of co-culture and fibroblast-conditioned medium (FCM) on clonal dynamics. (**a**) Relative growth of LS180 and HACAT colonies under co-culture and FCM conditions. Each data point represents the number of cells in a single colony on day 6 of the

experiment, normalized to the number of cells in the respective colonies at day 1 of the experiment. (**b**) Standard deviation of clone sizes in colonies analyzed in (**a**), representing clone growth heterogeneity. Each individual data point within the graph represents SD of a colony on day 6 of the experiment. These values are normalized based on the number of cells present in the corresponding colonies at day 1 of the experiment. (**c,d**) Changes in the clonal composition kinetics for co-culture and FCM experiments over time. Each graph displays every clone examined in the specified cell line. On these graphs, every line illustrates the percentage that a particular clone contributes to its own colony. (**e,f**) Alterations in clone sizes over time: increase, decrease, increase followed by decrease, decrease followed by increase, disappearance.

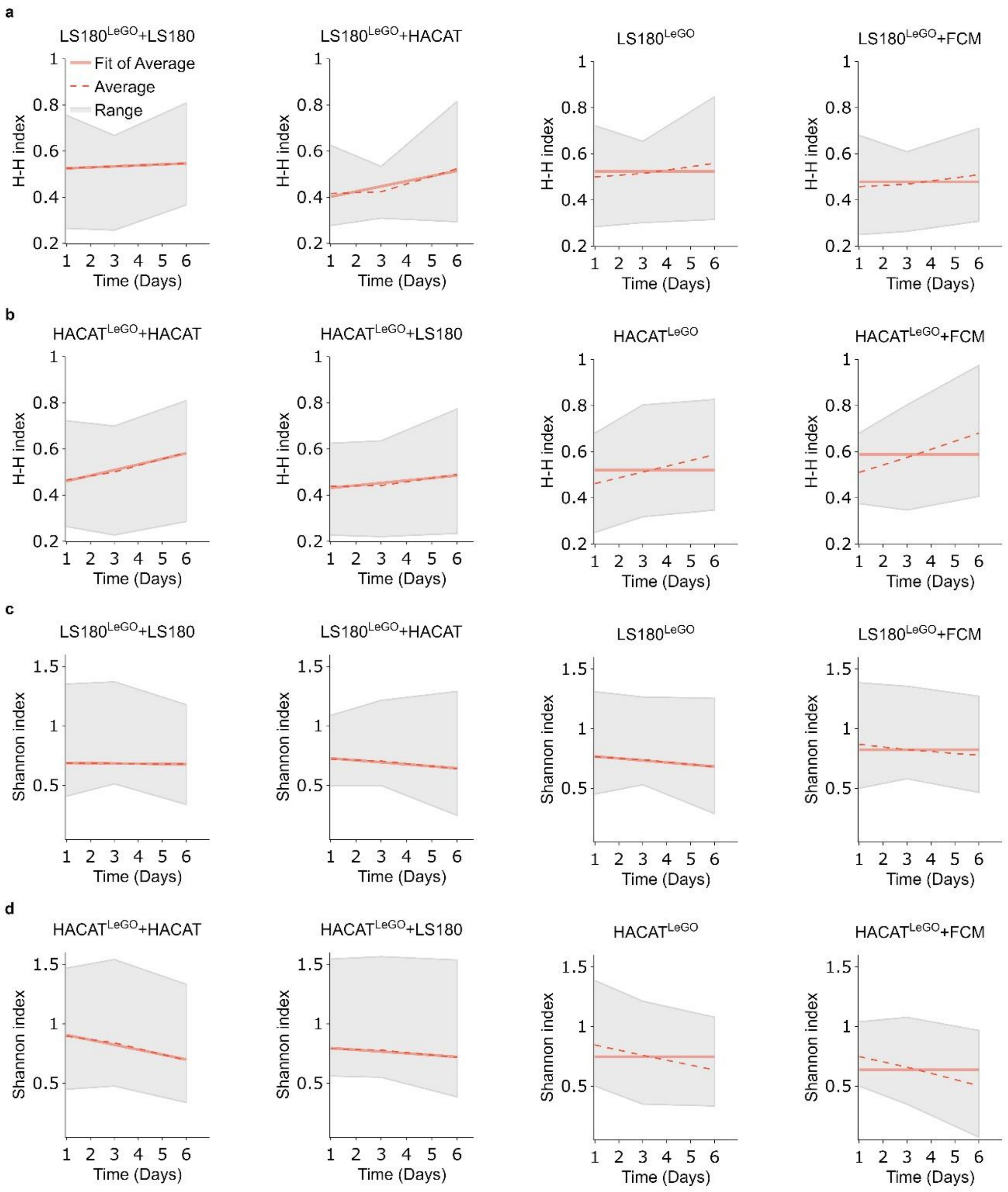

Supplementary figure 8. Clonal growth dynamics under co-culture and fibroblast-conditioned medium (FCM). (**a,b**) Changes in the Herfindahl-Hirschman index, a measure of clonal size dominance for the indicated cell lines, under co-culture and FCM conditions. The shaded areas represent the range of the index values, dotted and continuous lines represent the mean and linear-fitted values of Herfindahl-Hirschman index at the different timepoints, respectively. (**c,d**) Alterations in the Shannon index, a measure of clonal diversity for the indicated cell lines, under co-culture and FCM conditions. The shaded areas represent the range of the index values, dotted and continuous lines represent the mean and linear-fitted values of Shannon index at the different timepoints, respectively.
